# Functional Outcome Estimation of Bimalleolar Ankle Fractures Treated by Open Reduction and Internal Fixation at a Tertiary Care Center: A Descriptive Cross-sectional Study

**DOI:** 10.31729/jnma.5532

**Published:** 2020-10-31

**Authors:** Rajeev Dwivedi, Ankit Karki, Roshan Bhattarai, Badri Rijal

**Affiliations:** 1 Department of Orthopedics, Lumbini Medical College and Teaching Hospital, Tansen, Palpa, Nepal; 2 Department of Orthopaedics, National Trauma Centre, Kathmandu, Nepal

**Keywords:** *ankle fracture*, *pain*, *walking*

## Abstract

**Introduction:**

Ankle fractures account for about 9% of all fractures in adults. Open reduction and internal fixation is the preferred treatment for such injuries. However, surgery is not free of complications, and outcomes following surgery are not always satisfactory. Therefore, this study aimed to estimate the functional outcomes of bimalleolar ankle fractures treated by open reduction and internal fixation.

**Methods:**

This descriptive, cross-sectional study was carried out at a tertiary care center in the western region of Nepal among the patients with bimalleolar ankle fractures from March 2017 to August 2020 after approval from the Institutional review committee. Convenience sampling was done to reach the sample size. Twenty-nine cases were included in the study. Data were recorded in proforma and data analysis was done in the statistical package for social sciences (SPSS 16.0). The American Orthopedic Foot and Ankle Society (AOFAS) ankle-hindfoot score was used to assess the final outcome.

**Results:**

At the final evaluation mean AOFAS ankle-hindfoot score was 89.86 (±7.95). According to the AOFAS ankle-hindfoot score, there were 19 excellent (65.51%), six good (20.68%), and four fair (13.79%) results. Complications in the form of superficial infection were seen in four (13.79%) cases.

**Conclusions:**

Functional outcomes following surgical treatment of bimalleolar ankle fractures are mostly excellent to good and complications following surgery are few, therefore, surgery is a better option of treatment in bimalleolar ankle fractures.

## INTRODUCTION

Ankle fractures account for about 9% of all fractures in adults.^1,2^ Fractures around the ankle are intra-articular fractures. Therefore, in displaced and unstable injuries anatomical reduction and stable fixation mainly by open reduction and internal fixation is the preferred treatment.^2–4^

Outcomes in ankle fractures depend on many factors like severity of the injury, anatomical restoration of fracture, associated chondral and ligament injuries, post-operative rehabilitation programs and co-morbid conditions.^5^ A certain proportion of patients do not show satisfactory outcomes despite anatomical restoration of fractures by surgery.^6^ Moreover, surgery is not free of complications. Studies regarding evaluation of functional outcomes of ankle fractures following surgical treatment are few.

Therefore, this study aimed to estimate the functional outcomes of bimalleolar ankle fractures treated by open reduction and internal fixation.

## METHODS

This descriptive cross-sectional study was conducted at the Department of Orthopedics, Lumbini Medical College, and Teaching Hospital (LMCTH) from March 2017 to August 2020 for over three and a half years. This study was approved by the Institutional Review Committee (IRC -LMC 07-G/020) of LMCTH, Tansen, Palpa, Nepal. Written informed consents were taken from each of the participants.

The study population is patients who have been admitted to this institution with a diagnosis of ankle fractures. Hospital records including case files, operative details, and discharge records were reviewed to enroll the patients for study after due permission from concern authority. Patients, greater than 17 years of age with bimalleolar ankle fractures treated by open reduction and internal fixation were included in the study. Unimalleolar fracture, bimalleolar fractures with posterior malleolus fractures, fractures managed by nonoperative methods, compound fractures, patients with impaired sensation in lower limbs, and patients less than 6 months post-operative follow-up were excluded from the study. Age, sex, mechanism of injury, laterality, type of fracture as per Denis-Weber classification, type of surgery, type of implant used and postoperative complications were recorded. Patients who visited for follow up themselves and patients who return for follow-up after telephonic communication were examined and final outcomes were measured with validated the American Orthopedic Foot and Ankle Society (AOFAS) ankle-hindfoot score.^7^ The AOFAS ankle-hindfoot score system assesses the intensity of pain and functional disability and mainly includes nine aspects: pain 40 points, function 50 points which include, maximum walking distance (blocks), walking surfaces, gait abnormality, sagittal motion (flexion plus extension), hindfoot motion (inversion plus eversion), ankle-hindfoot stability (anteroposterior, varus-valgus), and alignment 10 points. The score had a maximum of 100 points (best possible outcome). The results are considered as excellent when the scores ranged from 90 to 100, good when between 80 and 89, fair when ranging from 70 to 79, and bad when below 70. At the final follow-up, anteroposterior, lateral, and mortise view X-rays of the ankle were evaluated for evidence of fracture union, maintenance of the ankle mortise, and the presence of post-traumatic arthritis of the ankle joint.

A total of 48 patients with bimalleolar fractures meeting the inclusion criteria were eligible to participate in the study. Out of that, 29 patients available for final follow up and evaluation were included in the study.

Convenience sampling was done and the minimum sample size was calculated using the formula,

$$\begin{array}{ll} \text{n} & \text{= }{\text{Z}}^{\text{2}}\times \text{p}\times \text{q}/{\text{d}}^{\text{2}}\\ & \text{= }{\left(1.64\right)}^{\text{2}}\times 0.09\times (1-0.09)/{\left(0.1\right)}^{\text{2}}\\ & \text{= }22.02 \end{array}$$

Where,

Z = 1.64 at 90% Confidence Interval.
p = prevalence of ankle fractures, taken as 9 % from previous studies (references 1-2).
q = 1-p
d = margin of error, 10% the minimum sample size was calculated 23.

Data were recorded in the proforma, then coded and entry was done in the statistical package for the social sciences (SPSS) version 16.0. The data were processed and analyzed by using simple descriptive statistics; in terms of percentage and frequency.

## RESULTS

At the final evaluation, mean AOFAS score was 89.86(±7.95) out of maximum 100. The average pain score was 34.14±5 out of 40, the average function score was 46.52 ±5.32 out of 50 and the average alignment score was 9.48±1.55 out of 10. Functional outcomes in different Denis-Weber fracture groups are shown (Table 1). Excellent to good outcomes were seen in 25(86.20%) cases, fair results were seen in four (13.79%) cases and no case had a poor outcome.

**Table 1:** Mean AOFAS scores and different grades of functional outcomes as per AOFAS score in different Denis-Weber fracture types.

Denis-Weber type	Number of cases n (%)	Mean AOFAS score	Functional outcome (AOFAS Score) n (%)			
			Excellent	Good	Fair	Poor
Type A	5 (100)	96.8 (±4.08)	5 (100)	0 (0)	0 (0)	0 (0)
Type B	14 (100)	89.79 (±8.19)	9 (64.28)	3 (21.42)	2 (14.28)	0 (0)
Type C	10 (100)	86.50 (±7.30)	5 (50)	3 (30)	2 (20)	0 (0)

The demographic characteristics of patients are shown (Table 2). Mean follow-up duration was 18.9 (8.98) months, with range of 6 to 36 months. The most common technique of fixation for medial malleolus fracture was modified tension band wiring with Kirschner wires (K-wires) and stainless steel wires in 20 cases. Double malleolar screws were used in five cases and double cannulated cancellous screws were used in four cases. For the lateral malleolus fractures, a reconstruction plate was used in 18 cases, one-third tubular plate in six cases, anatomical locking compression plate distal fibula in three cases, and modified tension band wiring with Kirschner wire (K-wire) and stainless steel wires in two cases. An Inter-fragmentary lag screw was used in 14 cases. Syndesmotic screw fixation was done in 15 cases; in all cases of Denis-Weber type C syndesmotic fixation was done. Syndesmotic screw removal was done at 12 weeks in all cases.

**Table 2:** Demographic characteristics of patients (n = 29).

Patients Characteristics		Findings
Age		40.10 (± 12) years
Male to Female ratio		1.9:1
Age of male patients		37.47 (± 11.10)years
Age of female patients		45.10 ( ±12.67)year
Side of injury	Right	18 (62.06%)
	Left	11 (37.93%)
Mechanism of injury	Twisting injury	14 (48.27%)
	Road traffic accident	8 (27.58%)
	Fall from height	7 (24.13%)
Denis-Weber type	Type A	5 (17.24%)
	Type B	14 (48.27%)
	Type C	10 (34.48%)

Surgery for removal of the implants was done in nine (31.03%) cases. No cases of nonunion, malunion and osteoarthritis were seen. Complications were seen in four (13.79%) cases as superficial wound infection, three on the medial side, and one on the lateral side which healed with antibiotics and wound care.

## DISCUSSION

In the present study overall functional outcome as per the AOFAS ankle-hindfoot score was good and the mean AOFAS score was 89.86 (±7.95) out of maximum 100. Most of the patients experienced no pain or occasional pain. Most of the cases had excellent to good outcomes. Less severe injury as per Denis - Weber's classification has better outcomes. In DenisWeber type A, all patients had excellent results; in type B, 12 (85.71%) out of 14 patients had excellent to good outcomes, and in type C eight (80%) out of 10 patients had excellent to good outcomes.

Surgical treatment in ankle fractures is usually reserved for unstable ankle injuries.^8^ In the present study, we have included bimalleolar fractures which are considered to be unstable injuries. There are various studies showing results in favor of surgical treatment similar to the present study. In a study by Dhoju et al,^9^ 32 patients with bimalleolar fractures treated by open reduction and internal fixation were evaluated with the AOFAS score; most of the patients had excellent AOFAS scores with a mean score of 90.56±10.92. Less severe injuries as per Denis-Weber classification had better outcomes than severe injuries though it was not statistically significant.

S. M. Verhage et al,^10^ evaluated 243 patients with ankle fractures following surgery, with a mean follow-up duration of 9.6 years. Excellent results were seen as per the AOFAS score. Overall mean AOFAS score was 95 and no significant differences between the three main AO groups were found. Mean AOFAS scores in AO 44A, B, C group were 95, 95, and 94 respectively.

In a study by Egol et al,^11^ 232 patients with unstable ankle fractures treated surgically were evaluated according to the AOFAS score, where 90% of the patients had ≥ 90% functional recovery. One year after surgery, patients did well, with most experiencing little or mild pain and few restrictions in functional activities. In a recent systematic review, it was concluded that surgical treatment is beneficial in achieving the anatomical reduction and rigid surgical fixation which could provide better protection against malunion, nonunion, and loss of reduction but conservative and surgical methods provide equivalent functional outcomes.^12^

In a study by Nilsson et al,^13^ fifty-four patients with ankle fractures were evaluated at 14 months and three years following surgery. They used Olerud-Molander Ankle Score (OMAS) for assessment, the median OMAS was 75 at the 14-month and 85 at the three-year follow-up. Pain, stiffness, and swelling were present among more than half of the patients and 40% reported instability and problems when using stairs. They concluded the subjective outcomes after three years of surgical intervention for ankle fractures is poorer than expected.

The present study has a few limitations, limited sample size as all eligible patients were not available for evaluation. The minimum follow-up duration was only six months; outcomes may change over a longer follow-up duration.

## CONCLUSIONS

Functional outcomes following surgical treatment of bimalleolar ankle fractures are mostly excellent to good, less severe injuries have better outcomes, and complications following surgery are few. Therefore, surgery is a better option of treatment for bimalleolar ankle fractures.

## Conflict of Interest

**None.**
